# N-Acetyl Cysteine improves the diabetic cardiac function: possible role of fibrosis inhibition

**DOI:** 10.1186/s12872-015-0076-3

**Published:** 2015-08-06

**Authors:** Cong Liu, Xiao-Zhao Lu, Ming-Zhi Shen, Chang-Yang Xing, Jing Ma, Yun-You Duan, Li-Jun Yuan

**Affiliations:** Department of Ultrasound Diagnostics, Tangdu Hospital, Fourth Military Medical University, #569 Xinsi Road, Baqiao District Xi’an, 710038 China; Department of Biochemistry and Molecular Biology, Fourth Military Medical University, Xi’an, China

**Keywords:** Diabetic cardiomyopathy, Fibrosis, Reactive oxygen species, N-acetyl-L-cysteine, Cardiac function

## Abstract

**Background:**

Diabetic cardiomyopathy is one of the leading causes of death in diabetes mellitus (DM) patients. This study aimed to explore the therapeutic implication of N-acetyl-L-cysteine (NAC, an antioxidant and glutathione precursor) and the possible underlying mechanism.

**Methods:**

Thirty five 12-week-old male C57BL/6 mice were included. Twenty-five diabetic mice were induced by intraperitoneal injection of streptozocin (STZ, 150 mg/kg, Sigma-Aldrich) dissolved in a mix of citrate buffer after overnight fast. Mice with a blood glucose level above 13.5 mmol/L were considered diabetic. As a non-DM (diabetic) control, mice were injected with equal volume of citrate buffer. The 25 diabetic mice were divided into 5 groups with 5 animals in each group: including DM (diabetes without NAC treatment), and 4 different NAC treatment groups, namely NAC1, NAC3, NAC5 and NAC7, with the number defining the start time point of NAC treatment. In the 10 non-DM mice, mice were either untreated (Ctrl) or treated with NAC for 5 weeks (NAC only). Echocardiography was performed 12 weeks after STZ injection. Heart tissue were collected after echocardiography for Hematoxylin Eosin (HE) and Trichrome staining and ROS staining. Cardiac fibroblast cells were isolated, cultured and treated with high glucose plus NAC or the vehicle. qPCR analysis and CCK-8 assay were performed to observe fibrotic gene expression and cell proliferation.

**Results:**

We found that both cardiac systolic function and diastolic function were impaired, coupled with excessive reactive oxygen stress and cardiac fibrosis 12 weeks after STZ induction. NAC significantly reduced ROS generation and fibrosis, together with improved cardiac systolic function and diastolic function. Strikingly, NAC1 treatment, which had the earlier and longer treatment, produced significant improvement of cardiac function and less fibrosis. In the cardiac fibroblasts, NAC blocked cardiac fibroblast proliferation and collagen synthesis induced by hyperglycemia.

**Conclusions:**

Our study indicates that NAC treatment in diabetes effectively protects from diabetic cardiomyopathy, possibly through inhibiting the ROS production and fibrosis, which warrants further clarification.

**Electronic supplementary material:**

The online version of this article (doi:10.1186/s12872-015-0076-3) contains supplementary material, which is available to authorized users.

## Background

Diabetes mellitus (DM) is one of the most common chronic diseases in nearly all countries, and by 2030 people with diabetes is expected to rise to 552 million [1]. It has become a fast-growing global problem with huge social, health, and economic consequences. Among the diabetic complications, diabetic cardiomyopathy (mainly manifested as two interconnected pathological processes: cardiac hypertrophy and fibrosis) is considered as one of the leading causes of death [2–5]. It is well established that diabetes increases oxygen stress, which have a causative role in cardiac dysfunction [6]. N-Acetylcysteine (NAC) is a thiol-containing radical scavenger and glutathione precursor. Several studies have demonstrated that antioxidant treatment using NAC may attenuate the myocardial damage by protecting cardiomyocyte and endothelium from cell death [7, 8]. Taken together, role of ROS in diabetic cardiomyopathy is still evasive, especially how ROS involved in the cardiac fibrosis.

This study aimed to explore the therapeutic implication of NAC and the possible underlying mechanism. We evaluated the efficacy of anti-oxidative NAC in preventing cardiac fibrosis and ventricular functional remodeling in the mouse model of STZ-induced diabetes at different time points. We found that early treatment of NAC in STZ induced diabetic mice resulted in better outcome, while later treatment produced less beneficial results, suggesting that diabetic cardiomyopathy is an irreversible process or alternatively NAC is incapable to reverse the pathological process. Mechanisitically, we found that NAC blocked hyperglycemia promoted induced cardiac fibroblast proliferation and myofibroblast differentiation via inhibition of ROS. Our study revealed an irreversible role of ROS in cardiac fibrosis and related cardiac dysfunction, shedding light on anti-oxidative therapy in protecting from diabetic cardiomyopathy.

## Methods

All experiments involving animals were performed in adherence with the Guide for the Care and Use of Laboratory Animals, and approved by the Fourth Military Medical University Committee on Animal Care.

### Diabetes model and treatments

**Fig. 1:**
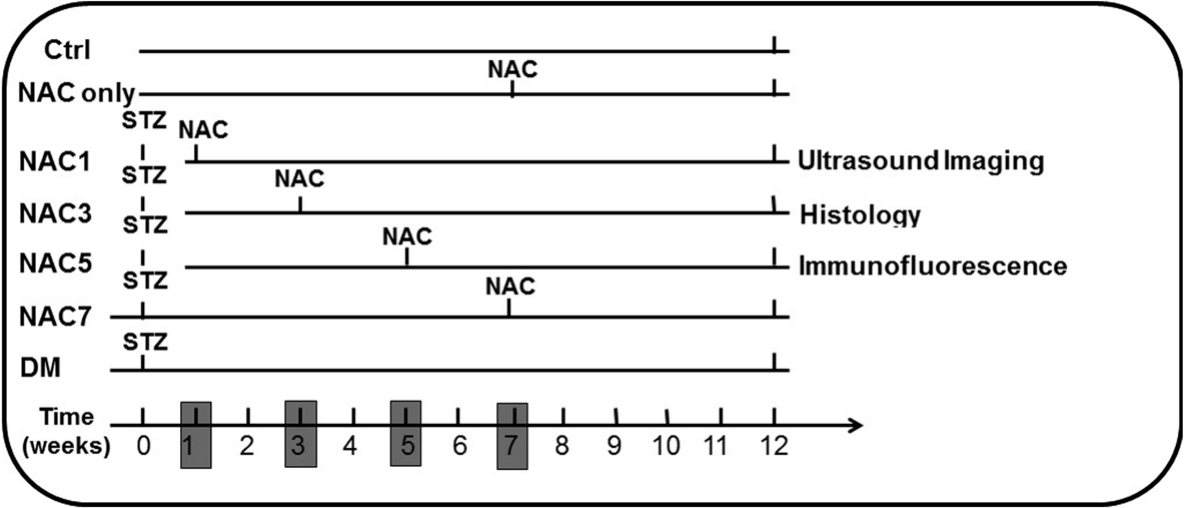
Schematic representation of the experimental procedure. Diabetic mouse model was induced by streptozotocin (STZ) injection. NAC treatment was done via drinking water starting from week 1, week 3, week 5 and week 7 STZ injection till the end of the week 12, respectively. Cardiac function and structure were analyzed by both echocardiography and histology

Twelve-week-old male C57BL/6 mice from the Experimental Animal Center of the Fourth Military Medical University were housed five/cage under a temperature of 25 ± 1 °C, 50 ± 5 % humidity, with an alternating 12 hrs light–dark cycle and free access to food and water ad libitum. The type of housing facility was specific pathogen free (SPF), and the cage is 30 cm (width) × 40 cm (depth) × 20 cm (height). For STZ induced diabetes model, mice were injected intraperitoneally with streptozocin (150 mg/kg, Sigma-Aldrich) dissolved in a mix of citrate buffer (citric acid and sodium citrate, pH 4.8) or vehicle (citrate buffer) after overnight fast similar as described before [9]. Blood glucose was checked 5 days later via tail vein; mice with a blood glucose level above 13.5 mmol/L were considered diabetic. As a control, mice were injected with equal volume of citrate buffer. In total, 35 mice were include in this study, which were divided into 7 groups with 5 animals in each group: including control, NAC only, DM (diabetes without NAC treatment), and 4 different NAC treatment groups. The 4 NAC treatment groups, namely NAC1, NAC3, NAC5 and NAC7, define the start time point when NAC treatments start. For example, in the NAC1 groups, diabetic mice were treated with NAC (A9165, Sigma-Aldrich) from 1 week after STZ induction at the dose of 1.0 g/kg body weight per day in drinking water. In the NAC only group, control mice were further treated with NAC for five weeks. No obvious adverse events were seen in each experimental group. The detailed procedure described in Fig. 1.

### Echocardiography

Echocardiography was performed from week 12 after STZ injection. Transthoracic 2-dimensional (2D), M-mode and Doppler echocardiographic studies were performed with Mylab 50 (Esaote, Italy) using a high-resolution transducer (SL3116) with frequency of 22 MHz. Briefly, each mouse was anesthetized by injecting intraperitoneally with 10 % chloral hydrate at the dose of 350 mg/kg body weight before echocardiographic study [10], which had an onset of sedation within 5–10 minutes and was maintained for about 30–40 minutes. Heart rates were monitored and generally maintained around 450 beats per minute. The chest hairs were removed using Depilatory creams. The mouse was then placed on a warm pad to keep the body temperature around 36 ± 0.5°C. Warmed echo gel was placed on the shaved chest as a coupling medium while the mouse lay on the warm pad at a supine position. Images were acquired and analyzed by an operator blinded to mouse treatment.

Interventricular septal thickness and LV posterior wall thickness during diastole (IVSd, LVPWd), LV internal dimensions during diastole (LVIDd) and systole (LVIDs) were measured from M-mode images at the level of the papillary muscles at LV short-axis view (Additional file 1: Figure S1a). Representative images were digitally acquired and stored on the internal hard disk and USB Mass Storage Device for off-line analysis. LV ejection fraction (EF), LV fractional shortening (FS) were calculated according to the recommendation of the American Society of Echocardiography Committee [11].

Transmitral inflow Doppler was obtained from the apical 4-chamber view. The sample volume was placed just below the level of the mitral annulus and adjusted to the position at which the velocity was maximal. The angle correction was kept less than 20 degree. LV diastolic function was evaluated using the methods described previously [12]. In brief, the left ventricular isovolumic relaxation time (IVRT) and the acceleration and deceleration times of the early peak (E) wave (E_AT_ and E_DT_, respectively) were derived respectively from the Doppler waveform (Additional file 1: Figure S1b).

### Tissue collection and histology

After echocardiography, the heart was excised from the chest, trimmed of atria and large vessels and weighed. Half of the hearts (in the long axis view) were formalin-fixed for Hematoxylin Eosin (HE) and Trichrome staining, while the other half were mounted with OCT directly for ROS staining. For histological analysis, excised hearts were washed with saline solution, placed in 10 % formalin, and embedded in paraffin. Then, 5-μm thick sections were prepared and stained with Masson Trichrome staining for detecting the myocardial fibrosis [13]. To determine myocardial ROS generation, dihydroethidium (DHE) staining was included by probing for the ROS on the 5-μm frozen myocardial sections [14].

### Cardiac fibroblast cell isolation and culture

Fibroblasts were isolated from the hearts of normal P7 (postnatal day 7) male C57/Bl6 mice similar as previously described [15]. Briefly, 3 hearts were isolated and vessels and atria were removed before transferred to 1 mL of collagenase buffer. In the buffer, the ventricles were quickly minced into small pieces and digested for about 1 hour. Cell suspension were filtered with 100 μm filter and then centrifuged. The cell pellet was re-suspended and plated on a T75 tissue-culture flask (Corning Corp) in full medium supplemented with 10 % of fetal bovine serum (HyClone) and antibiotic-antimycotic solution. Non-adherent cells were removed after overnight culture, and adherent cells were cultivated as cardiac fibroblast. Only fibroblasts at passage 1 to 5 were used for the following experiments.

### qPCR analysis

Cardiac fibroblast cells were cultured in the serum free medium containing either 5.5 mM (normal glucose, NG) or 25 mM glucose (HG) with 10 ng/ml TGFβ1 and without insulin for 24 hrs. In the HG group, cells were further added with control or NAC (5 mM). RNA was isolated with TriZOL (Invitrogen). Reverse transcription was performed with the Superscript III First Strand Synthesis kit (Invitrogen). SYBR Green Mix I (Takara) was used for amplification, and samples were run on an ABI7500 Instrument (AB, USA). Gapdh was used as internal control. 2− ΔΔCt method was used for analysis (n = 3). The primers are listed as follows: Gapdh forward, 5′-TGGCCTTCCGTGTTCCTACCC-3′, Gapdh reverse, 5′-AGCCCAAGATGCCCTTCAGTG-3′; Col1a1 forward, Col1a1 reverse, 5′-GGAATCCATCGGTCATGCTCT-3′; CTGF forward, 5′-CCACCCGAGTTACCAATGACA-3′, CTGF reverse, 5′-CTTGGCGATTTTAGGTGTCCG-3′.

### Cell proliferation assay

Cardiac fibroblast cells were seeded in 96-well plates at a density of 1.5 × 10^3^ cells per well and treated as indicated. Cell numbers were analyzed by Cell Counting Kit-8 (Sigma-Aldrich) at 450-nm absorbance.

### Statistical analysis

All data were expressed as mean ± SD. The mean data of six groups were compared with one-way ANOVA. The intra-and inter-observer variability were analyzed using 2-tailed Student’s t-test and linear regression analysis. A P-value < 0.05 was considered statistically significant.

## Results

### Anatomical weights and physiological parameters of mice in different groups

**Fig. 2:**
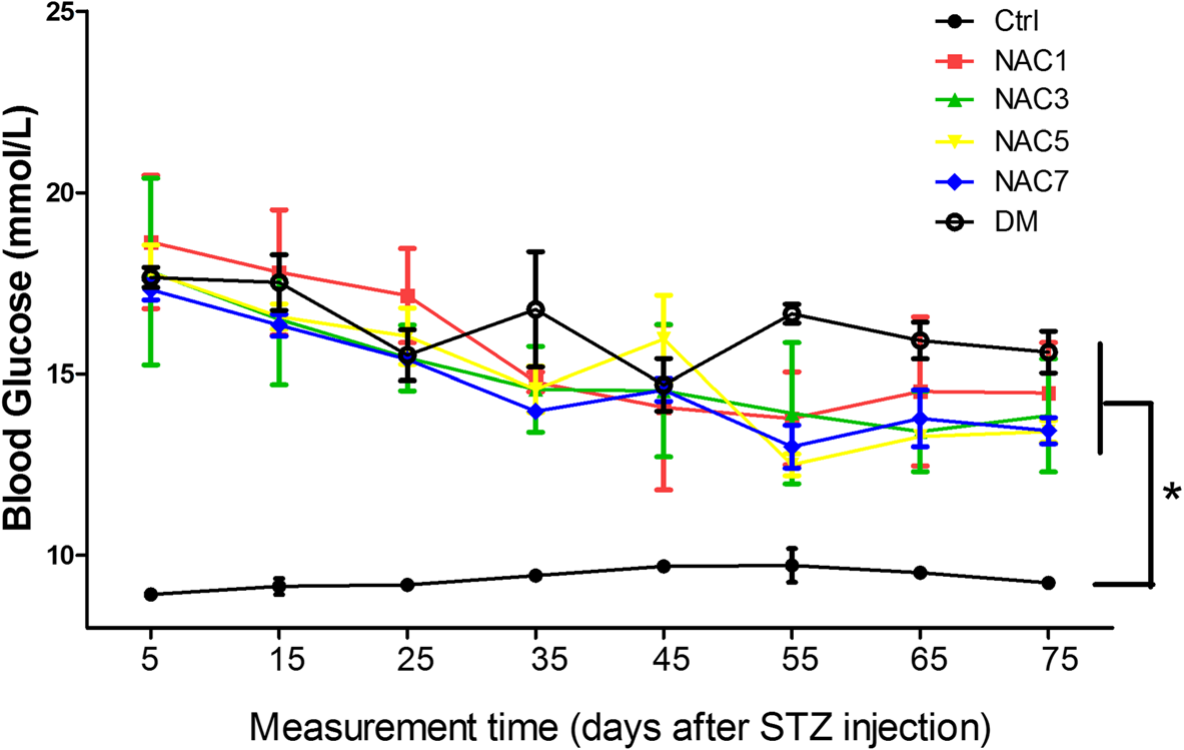
Blood glucose levels among all groups. Blood glucose levels of all the groups were compared and *denoted significant differences. (n = 5 in each group)

We first analyzed anatomical weights and other physiological parameters in mice with different treatments. As shown in Table 1, the body weight in diabetic group was much lower than that in the control group at the time of sacrifice. However there were no significant differences among all the diabetic mice either with or without NAC treatment. Similar as the body weight, all the diabetic mice either with or without NAC treatments had significant higher blood glucose levels than the control mice, while there were no significant differences among the NAC treatment groups (Fig. 2). All of these data suggest that NAC did not alter the body weight and blood glucose levels.

**Table 1:** Physiological parameters of mice in all groups

	Initial weight	Terminal weight	HW	HWI	HR
	(g)	(g)	(mg)	(mg/kg, %)	(bpm)
Control	24.32 ± 1.89	27.56 ± 2.33	168.12 ± 8.37	6.11 ± 0.22	446 ± 11
NAC only	23.44 ± 0.89	25.40 ± 0.54	160.40 ± 7.12	6.31 ± 0.21	448 ± 9
NAC1	23.22 ± 1.02	24.54 ± 0.65^*^	150.56 ± 7.07^*△^	6.10 ± 0.18^△^	445 ± 14
NAC3	23.67 ± 2.03	24.36 ± 1.91^*^	144.32 ± 13.42^*^	5.80 ± 0.25^*^	453 ± 21
NAC5	24.72 ± 1.31	24.45 ± 0.97^*^	144.00 ± 5.48^*^	5.78 ± 0.23^*^	440 ± 11
NAC7	24.86 ± 1.96	24.92 ± 1.83^*^	143.57 ± 8.94^*^	5.77 ± 0.14^*^	451 ± 19
DM	24.19 ± 1.60	24.84 ± 0.79^*^	138.00 ± 4.47^*^	5.56 ± 0.26^*^	441 ± 9
p value^a^	0.347	0.940	0.668	0.066	0.61

Notes: No significant differences were found between control and NAC only group; ^*^Compared with control, p < 0.05; ^△^NAC treatment groups compared with DM group, p < 0.05; No significant difference was seen in initial weight and heart rate among all groups. a, ANOVA test among the NAC1, NAC3, NAC5 and NAC7 four groups, no significant difference was seen among the four groups. HW, heart weight; HWI, heart weight index; HR, heart rate. (n = 5 in each group)

The heart weight and heart weight index in untreated DM group were significantly lower than in the control group, which was at least partially rescued by NAC treatment (Table 1). Notably, there was no significant decrease of HWI in NAC1 group compared with the control group.

### NAC treatments improve cardiac systolic and diastolic function in diabetic mice

The cardiac morphology and function differences among the 7 groups were measured by echocardiography and compared using ANOVA (Table 2).

**Table 2:** Comparison of echocardiographic morphological, systolic and diastolic functional indices

	IVSd	LVPWd	LVIDd	LVIDs	LVEF	LVFS	IVRT	E_DT_	E_AT_
	(mm)	(mm)	(mm)	(mm)	(%)	(%)	(ms)	(ms)	(ms)
Control	0.72 ± 0.04	0.70 ± 0.01	3.42 ± 0.31	1.98 ± 0.28	81.08 ± 4.6	42.31 ± 3.8	11.56 ± 1.74	11.00 ± 0.71	33.06 ± 3.27
NAC only	0.66 ± 0.05	0.67 ± 0.04	3.48 ± 0.08	2.06 ± 0.09	79.72 ± 2.58	40.80 ± 1.48	11.08 ± 0.66	11.80 ± 0.97	34.06 ± 2.48
NAC1	0.66 ± 0.05^△^	0.65 ± 0.05^△^	3.44 ± 0.05^△^	2.00 ± 0.70^△^	80.79 ± 2.3^△^	41.86 ± 1.6^△^	14.86 ± 0.78^*△^	13.86 ± 1.49^*△^	33.84 ± 2.67^**△**^
NAC3	0.66 ± 0.05^△^	0.65 ± 0.05^△^	3.62 ± 0.04^△^	2.12 ± 0.13^△^	79.80 ± 3.1^△^	41.45 ± 3.1^△^	15.00 ± 0.71^*△^	14.60 ± 0.89^*△^	34.24 ± 3.74^**△**^
NAC5	0.64 ± 0.05^*△^	0.59 ± 0.02^*^	3.92 ± 0.17^*△^	2.44 ± 0.15^*△^	75.89 ± 1.4^*△^	37.78 ± 1.2^*△^	18.14 ± 1.33^*△^	17.14 ± 2.02^*△^	35.56 ± 3.56^**△**^
NAC7	0.62 ± 0.04^*△^	0.56 ± 0.04^*^	4.10 ± 0.25^*△^	2.84 ± 0.21^*△^	66.69 ± 3.1^*△^	30.74 ± 2.2^*△^	20.74 ± 1.02^*^	20.38 ± 1.07^*^	37.18 ± 2.84
DM	0.52 ± 0.07^*^	0.53 ± 0.07^*^	4.76 ± 0.28^*^	3.58 ± 0.24^*^	57.21 ± 5.3^*^	24.75 ± 3.2^*^	21.08 ± 0.99^*^	21.22 ± 3.14^*^	48.60 ± 2.30^*^
p value^a^	0.585	0.008	<0.0001	<0.0001	<0.0001	<0.0001	<0.0001	<0.0001	0.384

Notes: No significant differences were found between control and NAC only group; ^*^Compared with control, p < 0.05; ^△^NAC treatment groups compared with DM group, p < 0.05;a, ANOVA test among the NAC1, NAC3, NAC5 and NAC7 four groups; IVSd and LVEPWd, interventricular septal thickness and left ventricular posterior wall thickness during diastole; LVIDd and LVIDs, left ventricular internal diameter during diastole and systole; LVEF, left ventricular ejection fraction; LVFS, left ventricular fractional shortening; IVRT, isovolumic relaxation time; E_DT_, descending time of the transmitral Doppler E wave; E_AT_ ,acceleration time of the transmitral Doppler E wave

Compared with the control group, NAC only group had similar cardiac function, indicating that NAC did not change the normal cardiac function (Table 2). As expected, DM group displayed much lower left ventricular ejection fraction (LVEF) and left ventricular fractional shortening (LVFS), indicating significant decrease of systolic function. Coupled with the decreased systolic function in diabetic group, LV diastolic function was also significantly reduced, as seen by the increased IVRT, E_AT_ and E_DT_ in DM group (Table 2). In addition, DM group displayed thinner IVSd and LVPWd, which was consistent with the lower HWI.

NAC supplement improved the LVFS and LVEF. Notably, there was even no significant difference between control and NAC1 and NAC3 groups, indicating that NAC1 and NAC3 treatments significantly improved cardiac systolic function in mice with diabetic cardiomyopathy.

Similar to the improved cardiac systolic function, NAC treatments also increased the diastolic function. The values of IVRT, E_AT_ and E_DT_ in NAC1 group were close to the control group, while those in NAC7 group were close to the DM group (Table 2).

### NAC treatments inhibit ROS production and cardiac fibrosis

**Fig. 3:**
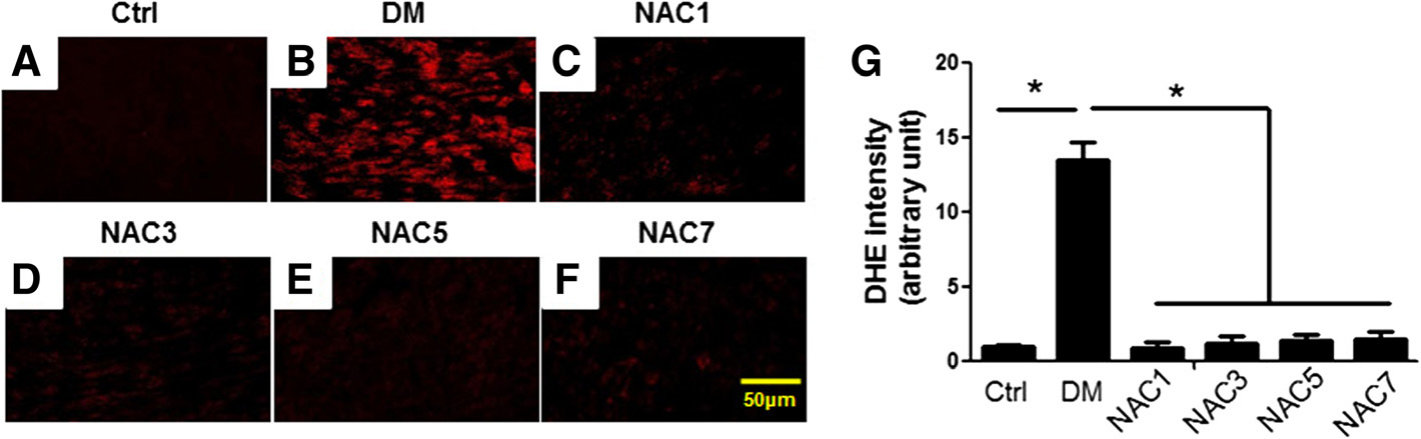
NAC reduces diabetic induced ROS in the heart. (**a**) DHE fluorescence of the heart section from the control mice. (**b**) DHE fluorescence of the heart section from the diabetic mice without NAC treatment. (**c-f**) DHE fluorescence of the heart section from the mice of NAC1 (**c**), NAC3 (**d**), NAC1 (**e**) and NAC3 (F) groups. Data presented are representative of the 5 mice in each group. (**g**) Quantification of the fluorescence intensity in the above groups

We next explored the mechanism how NAC improved the cardiac function, mainly focused on ROS generation and cardiac fibrosis. ROS generation was determined in the frozen section of the myocardial tissues by DHE fluorescence. Diabetic heart displayed robust increase of ROS all over the heart (Fig. 3a-b), which was efficiently cleared in all the NAC treatment groups (Fig. 3c-f). Quantification data were shown in Fig. 3g.

**Fig. 4:**
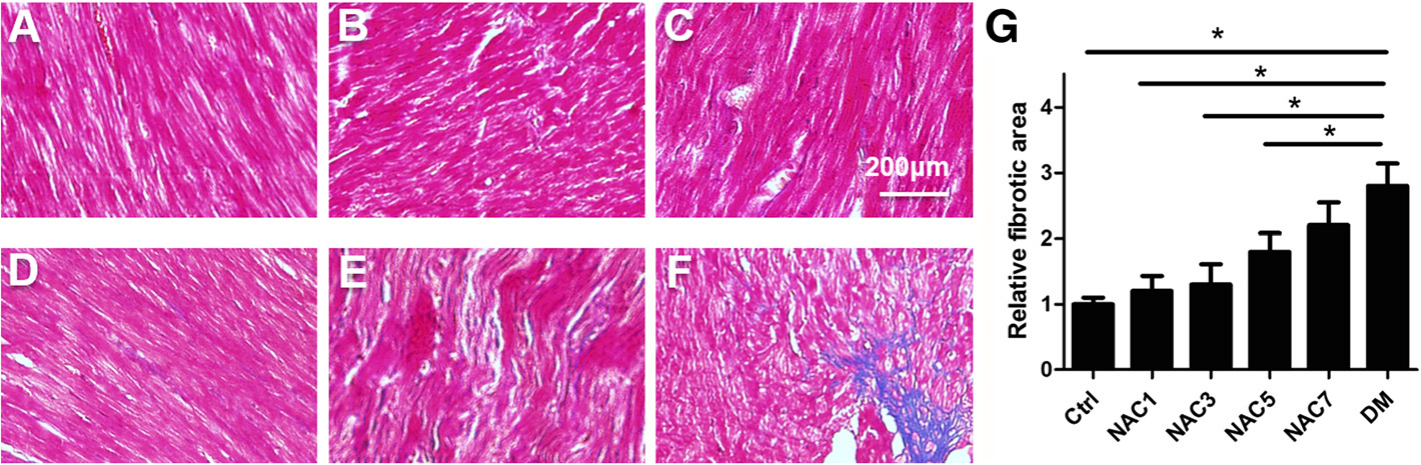
Masson Trichrome staining of interstitial fibrosis in different groups. (**a-f**) Masson Trichrome staining of the heart section from control (**a**), NAC1 (**b**), NAC3 (**c**), NAC5 (**d**), NAC7 (**e**) and DM (**f**) groups. Data presented are representative of the 5 mice in each group. (**g**) Quantification data of Figure a-f

Approximately 27 % of cells in the myocardium are fibroblasts [16], indicating that increased ROS in fibroblasts might be important in fibrosis and subsequent cardiac dysfunction. Consistent with the increase of NAC in DM group, Masson Trichrome staining revealed that the fibrotic area was significantly larger than that in the control group. NAC1 nearly attenuated the fibrosis induced by diabetes, while NAC3, NAC5 and NAC7 groups had weaker effects (Fig. 4a-g).

### NAC inhibits high glucose induced fibroblast proliferation and collagen synthesis

**Fig. 5:**
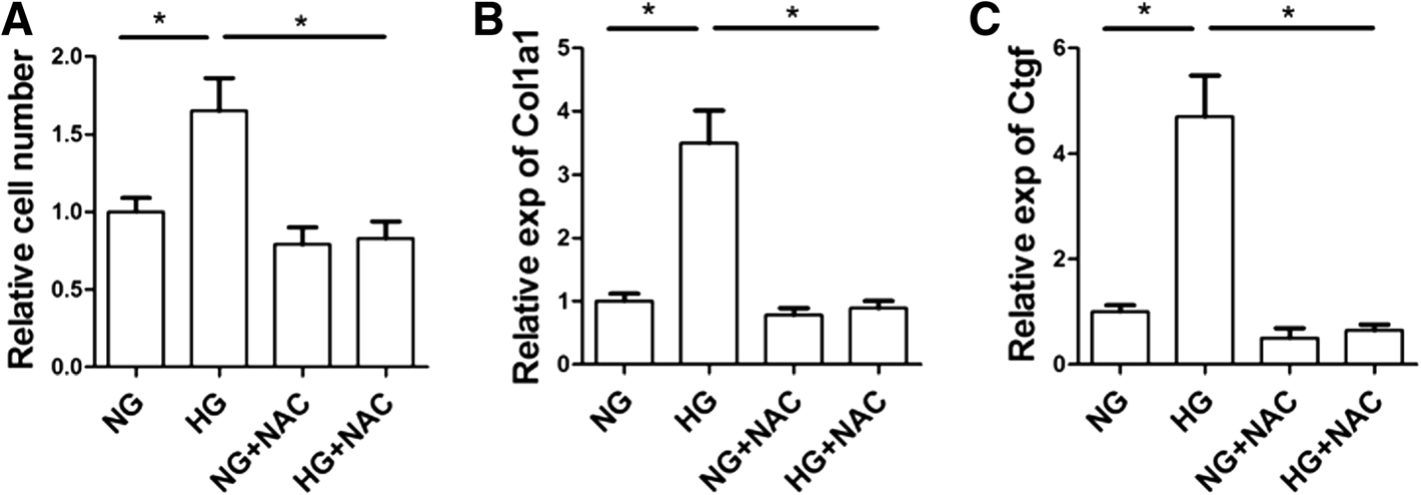
Effects of NAC on high glucose induced fibroblast proliferation and collagen expression. (**a**) Cardiac fibroblast cells were cultured in 5.5 mM glucose (NG) and 25 mM glucose (HG) w/o 5 mM NAC. Viable cell numbers were calculated by CCK-8 kit. *p<0.05, n=3. (**b**) Col1a1 gene expression in cardiac fibroblasts cultured in serum free medium with 10 ng/ml TGFβ1 containing 5.5 mM glucose, 25 mM glucose with/o 5 mM NAC. *p<0.05, n=3. (**c**) CTGF gene expression in cardiac fibroblasts treated same as above. *p<0.05, n=3

Per the overt effects of NAC on cardiac fibrosis, we next tested the role of NAC on cardiac fibroblast proliferation and collagen synthesis. Isolated cardiac fibroblast cells were cultured in normal and high glucose medium. Compared with the normal glucose medium, high glucose slightly stimulated the fibroblast proliferation, which was totally blocked by NAC treatment (Fig. 5a). Besides the pro-proliferative role of high glucose on cardiac fibroblasts, high glucose also increased TGFβ1 induced expression of Col1a1 and CTGF (Fig. 5b-c). Again, NAC attenuated the Col1a1 and CTGF expression induced by TGFβ1.

## Discussion

By using the STZ induced diabetes mouse model, we systematically analyzed the function of NAC, a potent inhibitor of ROS, in preventing cardiac dysfunction. To our knowledge, we for the first time revealed that: earlier and longer NAC treatment improves both the systolic and diastolic function, which is coincided with the reduced ROS generation and fibrosis.

It has been suggested that oxidative stress plays a critical role in inducing cardiomyopathy and heart failure in chronic diabetes [17–19]. Oxidative stress results from an imbalance between the generation of oxygen derived radicals and the organism’s antioxidant potential [20].Various studies have shown that DM is associated with increased formation of free radicals and decrease in antioxidant potential. Increased oxidative stress has been suggested to be a common pathway linking diverse mechanisms for the pathogenesis of complications in diabetes [21, 22]. Previous studies have showed that increased ROS could lead to apoptosis in both endothelial cells and cardiomyocytes [8, 23], which are considered as the main causes of cardiac dysfunction. In fact, both endothelial and cardiomyocyte apoptosis would result in fibrosis, a kind of remodeling and repair. Our study here also found that ROS could directly promote fibrosis via promoting fibroblast proliferation and collagen synthesis in the setting of diabetes. In fact, the role of ROS in fibroblast activation has been found in the diseased prostatic stroma and other systems [24], further strengthening the role of ROS activated fibroblast in cardiac dysfunction. However, it is unknown how much fibrosis contributes to the diabetic cardiomyopathy, especially when endothelial cell and cardiomyocyte apoptosis is considered. It is highly possible that endothelium and cardiomyocyte apoptosis, and fibroblast activation form a viscous cycle that resulting in cardiomyopathy. And targeting the viscous is of therapeutic potential.

In this study, we found that NAC1 treatment group improved cardiac function much more than other NAC groups. Although previous studies have documented that NAC treatment suppressed ventricular structural and functional remodeling of the DM cardiomyopathy [5, 8]. Besides the evasive cardiac protection mechanism of NAC in the diabetic mice talked above, the time window of NAC action is unknown. The current study demonstrates that NAC 1 treatment, which NAC supplementation is earlier and longer results in the better cardiac outcomes than any other NAC treatment groups. One of the explanation is that toxic ROS generation begins and persists as early as onset of diabetes. In that case, longer and earlier NAC treatment would be beneficial.

### Limitations

Notably, there are several limitations to this study. First, although we stress the importance of ROS in fibroblast and fibrosis, we still don’t know how much the ROS-fibrosis contributes to the myocardiopathy in diabetes, especially their roles relative to the effects of ROS on cardiomyocytes and endothelial cells. Future studies by using fibrosis inhibitors would produce more informative data. Secondly, we haven’t clarified the earlier or longer treatment of NAC in NAC1 group is better. Future studies comparing treatments with the same duration and different starting time point would possibly answer the question. Thirdly, we don’t know whether NAC has a therapeutic function besides the preventive effects [17].

## Conclusions

Increase of ROS plays an important role in the development of the ventricular remodeling and cardiomyopathy in the setting of diabetes. NAC treatment in diabetes effectively improves the cardiac function, either by preventing from diabetic cardiomyopathy or rescuing the cardiac function, which needs further clarification.

## Additional file

Additional file 1:
**Figure S1.** Representative image describing how cardiac function is evaluated by echocardiography. (A) Representative M-mode image of control mouse exemplifying left ventricle contraction function measurements under short-axis view. VSd and LVPWd, intervenItricular septal thickness and left ventricular posterior wall thickness during diastole; LVIDd and LVIDs, Left ventricular internal diameter during diastole and systole. (B) Pulsed-wave Doppler of transmitral inflow image of control mouse exemplifying left ventricular diastolic function measurements. E, peak early diastolic transmitral Doppler flow velocity, IVRT, isovolumic relaxation time; E_AT_ and E_DT_, acceleration time and deceleration time of E wave. (TIFF 530 kb)

## Abbreviations

DM: Diabetes Mellitus
ROS: Reactive Oxygen Species
NAC: N-Acetyl-L-Cysteine
STZ: Streptozocin
HE: Hematoxylin Eosin
IVSd: Interventricular Septal Thickness during Diastole
LVPWd: Left Ventricular Posterior Wall Thickness during Diastole
LVIDd: Left Ventricular Internal Dimensions during Diastole
LVIDs: Left Ventricular Internal Dimensions during Systole
LVEF: Left Ventricular Ejection Fraction
LVFS: Left Ventricular Fractional Shortening
IVRT: Isovolumic Relaxation Time
E: Peak Early Diastolic Transmitral Doppler Flow Velocity
E_AT_: Acceleration Time of E wave
E_DT_: Deceleration Time of E Wave
DHE: Dihydroethidium
HG: Glucose
HWI: Heart Weight Index
