# Supplementary figures and images for: N-Acetyl Cysteine improves the diabetic cardiac function: possible role of fibrosis inhibition
Source: BMC Cardiovasc Disord. 2015 Aug 6;15:84. doi: 10.1186/s12872-015-0076-3 (PMC4525750; doi:10.1186/s12872-015-0076-3)

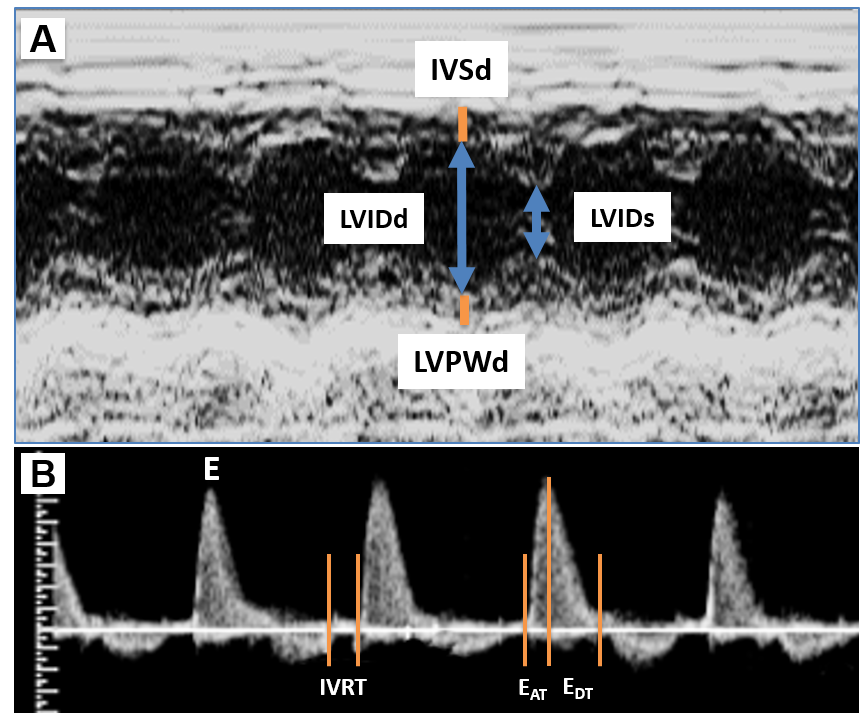

Supplement: Additional file 1: — Figure S1. Representative image describing how cardiac function is evaluated by echocardiography. (A) Representative M-mode image of control mouse exemplifying left ventricle contraction function measurements under short-axis view. VSd and LVPWd, intervenItricular septal thickness and left ventricular posterior wall thickness during diastole; LVIDd and LVIDs, Left ventricular internal diameter during diastole and systole. (B) Pulsed-wave Doppler of transmitral inflow image of control mouse exemplifying left ventricular diastolic function measurements. E, peak early diastolic transmitral Doppler flow velocity, IVRT, isovolumic relaxation time; EAT and EDT, acceleration time and deceleration time of E wave. (TIFF 530 kb) [file 12872_2015_76_MOESM1_ESM.tiff]
